# E3 ligase UHRF2 hijacks nuclear TBK1 to epigenetically repress type I interferons expression

**DOI:** 10.7150/ijbs.135125

**Published:** 2026-05-18

**Authors:** Wenwen Huang, Renjie Song, Qicong Shen, Yang Shi, Xi Wang, Nan Li, Qian Zhang, Xuetao Cao

**Affiliations:** 1 Institute of Immunology, Zhejiang University School of Medicine, Hangzhou 310058, China.; 2 National Key Laboratory of Immunity and Inflammation, Institute of Immunology, Naval Medical University, Shanghai 200433, China.; 3 Department of Immunology, Institute of Basic Medical Sciences & School of Basic Medicine, Chinese Academy of Medical Sciences & Peking Union Medical College, Beijing 100005, China.

**Keywords:** type I interferons, antiviral immunity, epigenetic regulation, histone lactylation, UHRF2

## Abstract

The rapid induction of type I interferons (IFN-I) by innate signaling is indispensable for host defense; however, its uncontrolled expression invariably leads to autoimmune diseases. Here, we reveal that the E3 ubiquitin ligase UHRF2 functions as a highly specific epigenetic repressor of IFN-I gene transcription. During viral infection, activated TBK1 undergoes nuclear translocation, where it is hijacked by UHRF2 to achieve gene-specific targeting at IFN-I loci. Upon recruitment to IFN-I loci, UHRF2 physically interacts with histone deacetylase 1 (HDAC1), catalyzes atypical K29-linked polyubiquitination and prevents HDAC1 from degradation. This stabilized UHRF2-HDAC1 complex actively erases the lactylation of histone H4 at lysine 12 (H4K12la), thereby silencing IFN-I transcription. To mount an effective initial antiviral response, IFN-I signaling feedback transiently downregulates UHRF2 expression. Consequently, *UHRF2*-deficient mice exhibit profound resistance to lethal virus infection in vivo due to IFN-I overproduction. These findings uncover a highly coordinated mechanism wherein nuclear TBK1, UHRF2, and HDAC1 converge to epigenetically regulate immune homeostasis through histone delactylation, identifying UHRF2 as a potential therapeutic target for virus infection or autoimmune diseases.

## Introduction

Type I interferons (IFN-I) play central roles in antiviral immunity, as well as serve as the critical coordinators in autoimmunity and cancer immune surveillance 1, 2. The signaling cascades culminating in IFN-I transcription are initiated by the engagement of pattern recognition receptors (PRRs), including Toll-like receptors (TLRs), RNA sensor like retinoic acid inducible gene-I (RIG-I)-like receptors, and DNA sensors such as cyclic GMP-AMP synthase (cGAS) and heterogeneous nuclear ribonucleoprotein A2B1 (hnRNPA2B1) 3-5. Upon the detection of viral nucleic acids, these sensors recruit specific adaptor proteins that converge on the activation of TANK-binding kinase 1 (TBK1) and its homolog IκB kinase epsilon (IKKε). TBK1, a versatile serine/threonine kinase, plays a non-redundant role in phosphorylating the transcription factors interferon regulatory factor 3 (IRF3) and IRF7, inducing INF-I transcription 6, 7. While the rapid induction of IFN-I is crucial for eliminating the invading pathogens, the magnitude and duration of this response must be precisely regulated 8. Chronic or excessive IFN-I production is a primary driver of autoinflammatory and autoimmune disorders, necessitating the existence of sophisticated negative regulatory networks.

The epigenetic regulation of innate immunity has gained prominence as a mechanism for determining cell-specific phenotypes and transcriptional memory during infection 9-11. For the epigenetic regulation of IFN-I expression, DNA methylation and histone methylation are reported to either indirectly regulate the regulators of antiviral signaling or directly regulate transcription of IFN-I 12, 13. Beyond classical DNA methylation and histone acetylation, the intersection of cellular metabolism and epigenetics has recently yielded the discovery of histone L-lactylation (Kla) 14. L-lactylation of histone tails facilitate the robust transcription of target genes, including those critical for the inflammatory response 15. The removal of these lactyl marks is essential for transcriptional silencing and the resolution of inflammation. Recently, class I histone deacetylases, particularly HDAC1, HDAC2, and HDAC3, have been identified as the primary endogenous delactylases responsible for erasing these metabolism-driven activating marks 16. How these epigenetic erasers are specifically recruited to and stabilized at IFN-I loci during the resolution phase of viral infection remains poorly understood.

Within this epigenetic framework, the ubiquitin-like containing PHD and RING finger domains (UHRF) proteins emerge as a compelling candidate for bridging signaling cascades with epigenetic silencing. UHRF1 recognizes hemimethylated DNA and recruits DNMT1 for maintaining DNA methylation during mitosis 17, Although UHRF2 shares same domains as UHRF1 but seems not implicated in general maintenance methylation due to no preference for hemimethylated DNA 18, 19. UHRF2 is reported to be a reader of 5-hydroxymethylated DNA through its SRA domain 20, 21. Upon reading 5-hydroxymethylated DNA, UHRF2 acts as a ubiquitin E3 ligase to catalyze K33-linked polyubiquitination of XRCC1 to promote accomplishment of active DNA demethylation 22. UHRF2 also interacts with other chromatin modifiers to regulate chromatin modifications like histone acetylation 23-25. Despite its known role as an epigenetic hub, the involvement of UHRF2 in the innate immunity, and specifically its capacity to govern the chromatin states and regulate innate immune response is still unclear.

In this study, we identify UHRF2 as a master epigenetic repressor of the IFN-I production. We delineate a previously unrecognized mechanism wherein UHRF2 physically interacts and hijacks nuclear-translocated TBK1, utilizing the kinase as a molecular beacon to locate and bind the IFN-I gene loci. At the chromatin level, UHRF2 orchestrates the atypical K29-linked polyubiquitination of HDAC1, enhancing the stability of the HDAC1 protein. Consequently, the stabilized HDAC1 actively erases L-lactylation of histone H4 lysine 12 (H4K12la) at the IFN-I loci, precipitating the epigenetic shutdown of antiviral transcription. Furthermore, we reveal that the IFN-I pathway transiently suppresses UHRF2 expression during the early stages of viral infection, establishing a temporal positive feedback loop that licenses maximal initial interferon production before UHRF2-mediated repression becomes the dominant process.

## Results

### UHRF2 is downregulated by type I interferon signaling to fuel the expression of IFN-I

**Figure 1:**
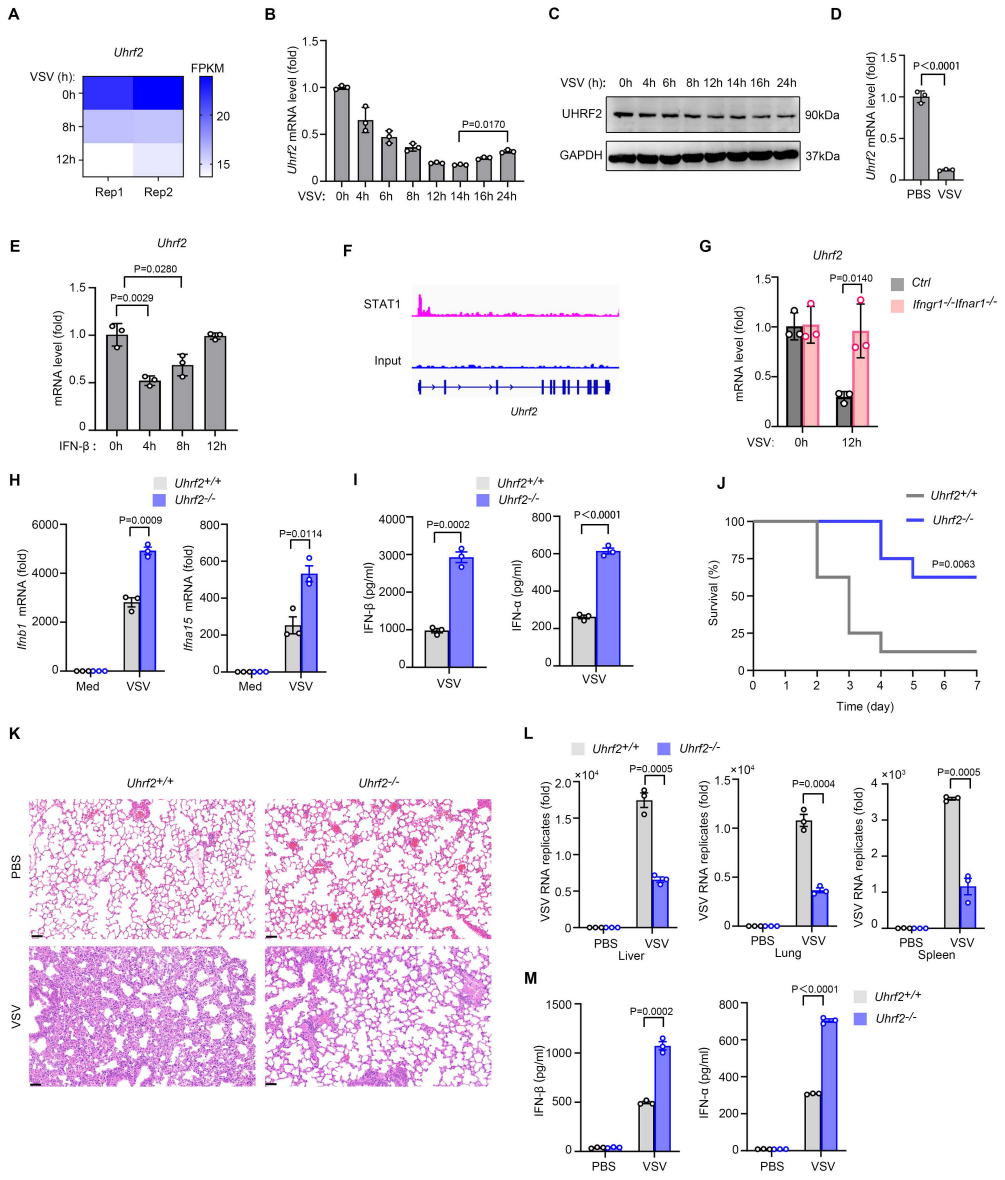
**IFN-I signaling inhibits UHRF2 expression to fuel the expression of IFN-I against viral infection.** (**A**) RNA-seq analysis of *Uhrf2* expression levels in mouse macrophages after VSV infection for the indicated time. (**B**) RT-qPCR analysis of *Uhrf2* mRNA level in macrophages after VSV infection for the indicated time, normalized by *Gapdh* mRNA. (**C**) Immunoblot analysis of UHRF2 and GAPDH in macrophages after VSV infection for the indicated time. (**D**) RT-qPCR analysis of *Uhrf2* mRNA levels in lung macrophages isolated from mice infected with VSV *in vivo* for 12 h. (**E**) RT-qPCR analysis of *Uhrf2* mRNA levels in macrophages stimulated with IFN-β for the indicated time. (**F**) STAT1 ChIP-seq signals at the promoter region of *Uhrf2* gene in activated mouse macrophages. (**G**) RT-qPCR analysis of *Uhrf2* mRNA levels in macrophages from *Ifngr1^-/-^Ifnar1^-/-^* mice infected with or without VSV for 12h. (**H**) RT-qPCR analysis of mRNA levels of indicated genes in *Uhrf2*^+/+^ or *Uhrf2*^-/-^ macrophages infected with VSV for 12 h. (**I**) ELISA of IFN-β and IFN-α of *Uhrf2^+/+^* or *Uhrf2^-/-^* macrophages infected with VSV for 12 h. (**J**) Kaplan-Meier survival of *Uhrf2^+/+^* or *Uhrf2^-/-^* mice (n=8 per group) after intraperitoneal injection of VSV (5 × 10^7^ plaque-forming units per gram body weight). (**K**) Hematoxylin-and-eosin staining of sections of lungs from *Uhrf2^+/+^* or *Uhrf2^-/-^* mice 24 h after intraperitoneal injection of PBS or VSV. Scale bars, 100 μm. (**L**) RT-qPCR analysis of VSV RNA in the liver, lung and spleen of *Uhrf2^+/+^* or *Uhrf2^-/-^* mice 24 h after intraperitoneal injection of PBS or VSV (as in **K**). (**M**) ELISA of IFN-β and IFN-α in serum of *Uhrf2^+/+^* and *Uhrf2^-/-^* mice infected with VSV for 24 h. Data were presented as mean+s.d (**B, D, E, G, H, I, L, M**) or images (**C**) from one representative of three independent experiments with biological replicates, or a minimum sample size of 3 per group (**K, L, M**).

To identify novel epigenetic regulators involved in the innate antiviral response, we retrospectively analyzed published RNA-sequencing (RNA-seq) datasets tracking the transcriptomic alterations in murine macrophages infected with vesicular stomatitis virus (VSV) 26. We found that the mRNA levels of UHRF2 were decreased during VSV infection (**Fig. 1A**). We further validated decreased both mRNA and protein levels of UHRF2, but a gradual restoration of UHRF2 levels at later time points in peritoneal macrophages during VSV infection (**Fig. 1B, C**). The rise of protein levels of UHRF2 at late time points during infection were not observed, probably due to delayed change of protein levels. To ensure that this phenomenon was not merely an artifact of cell culture conditions, we extended our analysis to an in vivo model, observing a parallel suppression of UHRF2 mRNAs in macrophages in lung from mice subjected to VSV infection for 12 hours (**Fig. 1D**).

The transient downregulation of UHRF2 suggested it might be actively repressed by the cytokines induced during the initial viral detection. Cross-referencing the database *INTERFEROME,* which focuses on interferon-regulated genes 27, proposed UHRF2 as a potential interferon-repressed target. Treatment of peritoneal macrophages with recombinant IFN-β rapidly and transiently inhibited UHRF2 mRNA expression, mirroring the kinetics observed during live viral infection (**Fig. 1E**). Mechanistically, analysis of publicly available chromatin immunoprecipitation sequencing (ChIP-seq) data (GSE176146) 28 identified robust binding of the transcription factor STAT1 to the promoter region of the *Uhrf2* gene in activated macrophages (**Fig. 1F**). STAT1 is widely known for driving the transcription of ISGs, but it also functions as a potent transcriptional repressor in specific genomic contexts 29. The absolute requirement for this autocrine/paracrine signaling loop was confirmed utilizing macrophages derived from Ifnar1-knockout mice, which lack a functional IFN-I receptor. In the absence of intact IFN-I signaling, VSV infection entirely failed to downregulate UHRF2 (**Fig. 1G**). Collectively, these results indicate that UHRF2 is an interferon-repressed gene, suggesting that its downregulation is a prerequisite for relieving baseline epigenetic repression and allowing the early amplification of the antiviral response.

To functionally characterize the role of UHRF2 in antiviral immunity, we established *Uhrf2*-knockout (*Uhrf2*-KO) mice model using CRISPR-Cas9 technology. The loss of UHRF2 not perturb normal macrophage lineage commitment or resting cellularity in the peritoneal cavity, ruling out broad developmental defects (**Fig. S1A-D**). However, upon in vitro challenging with VSV, *Uhrf2*-KO macrophages exhibited a hyper-induction of both IFN-β and IFN-α mRNAs, accompanied by significantly elevated secretion of IFN-β and IFN-α proteins (**Fig. 1H, I**). Interestingly, the expression of classic proinflammatory cytokines, including TNFα, IL-1β and IL-6, were barely affected by UHRF2 loss (**Fig. S1E**), indicating this regulatory effect was highly specific to the interferon pathway.

The physiological consequence of this specific IFN-I hyper-response was starkly evident during in vivo viral challenge. *Uhrf2*-KO mice infected with a lethal dose of VSV demonstrated profoundly enhanced survival rates compared to their wild-type littermates (**Fig. 1J**). Pathological assessment via Hematoxylin-and-eosin (H&E) staining revealed significantly mitigated tissue damage and inflammatory infiltration in the lungs of the knockout animals (**Fig. 1K**). Furthermore, RT-qPCR analysis demonstrated a precipitous drop in viral RNA titers across multiple organs, including the liver, lung, and spleen, in the *Uhrf2*-deficient mice (**Fig. 1L**). This protective phenotype was directly attributable to the systemic overproduction of type I interferons in the knockout mice (**Fig. 1M**), demonstrating that UHRF2 functions as a highly specific, non-redundant negative regulator of IFN-I expression during the innate immune response.

### UHRF2 specifically represses IFN-I gene activity in a TBK1-dependent manner

**Figure 2:**
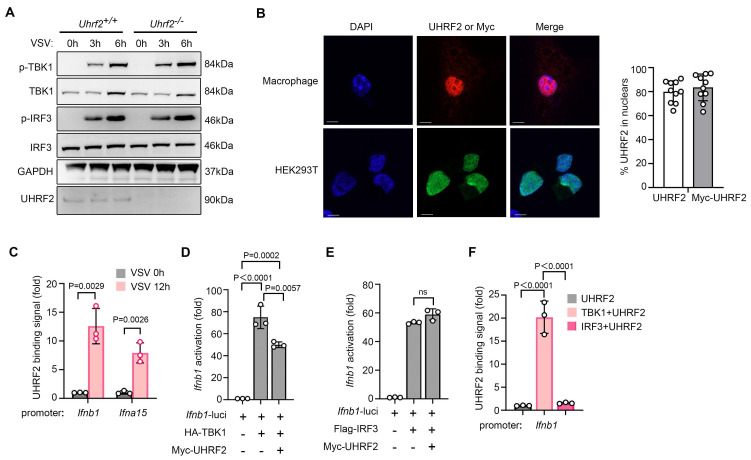
**TBK1 is required for UHRF2-mediated gene-specific repression of IFN-I.** (**A**) Immunoblot analysis of phosphorylated (p-) or total TBK1, IRF3, and UHRF2 or GAPDH in *Uhrf2^+/+^* or *Uhrf2^-/-^* mouse macrophages infected with VSV for various times. (**B**) Confocal microscopy of the subcellular location of endogenous UHRF2 in macrophages (top row) or overexpressed Myc-tagged UHRF2 in HEK293T cells (bottom row). DAPI, DNA-binding dye. Scales bar, 5 μm. Right panel: Quantification of UHRF2 nuclear enrichment (% cells, n = 10 cells per group). (**C**) ChIP-qPCR analysis of UHRF2 at the *Ifnb1* and *Ifna15* promoters in macrophages infected with VSV for 12 h. (**D, E**) Luciferase activity of an *Ifnb1* promoter reporter in HEK293T cells overexpressed with TBK1 or IRF3 with or without UHRF2, assessed by dual-luciferase assay. (**F**) ChIP-qPCR analysis of UHRF2 at the *Ifnb1* promoter in reporter in HEK293T cells overexpressed with indicated proteins. Data were presented as mean+s.d (**C, D, E, F**) or images (**A, B**) from one representative of three independent experiments with biological replicates.

We went further to investigate the mechanism underlying UHRF2 inhibition of IFN-I expression. We sought to determine the molecular node targeted by this epigenetic regulator. Initially, we hypothesized that UHRF2 might regulate the stability or activation of upstream cytosolic signaling components, such as sensors or kinases, which are frequent targets of E3 ligases. However, tracking the phosphorylation cascade revealed that the loss of UHRF2 only marginally enhanced the phosphorylation of TBK1 and IRF3 following VSV infection, which was insufficient to explain the profound magnitude of the transcriptional hyper-response observed at the mRNA level of IFN-I (**Fig. 2A**). Furthermore, confocal immunofluorescence microscopy revealed that the endogenous UHRF2 in primary macrophages was significantly enriched in the nucleus, and also the ectopically expressed UHRF2 in HEK293T cells was almost exclusively localized in the nucleus (**Fig. 2B**), indicating that UHRF2 is physically compartmentalized away from the primary cytosolic viral sensors and early kinase complexes. Thus, we proposed that UHRF2 directly regulates IFN-I expression in a gene-specific manner in nucleus.

The strict nuclear localization strongly implied that UHRF2 exerts direct and localized epigenetic control at the chromatin level. Using chromatin immunoprecipitation with quantitative PCR (ChIP-qPCR), we assessed the physical presence of UHRF2 at specific genomic loci. We found that in resting cells, UHRF2 occupancy at the *Ifnb1* and *Ifna15* promoters was minimal; however, following VSV infection, UHRF2 was rapidly and heavily recruited to the specific promoter regions of both *Ifnb1* and *Ifna15* (**Fig. 2C**). Because UHRF2 lacks a canonical sequence-specific DNA-binding domain 19, 20, we postulated that it must depend on the activation of anti-viral signaling pathways to guide it to the correct genomic address. To test this, we then performed luciferase reporter assays utilizing *Ifnb1* promoter. The ectopic overexpression of UHRF2 potently suppressed promoter activity that had been artificially stimulated by the overexpression of TBK1 but not IRF3 (**Fig. 2D, E**). Thus, TBK1 but not IRF3 may be required for specific targeting of UHRF2 to IFN-I genes. Indeed, in a reconstituted cellular system, overexpression of TBK1 was sufficient to induce the robust binding of UHRF2 to the *Ifnb1* promoter in a reporter context, whereas the overexpression of IRF3 was not (**Fig. 2F**). These data indicate that UHRF2 specific targets the promoters of IFN-I genes upon anti-viral signaling activation in a TBK1-dependent manner, and also suggest UHRF2 requires the specific structural or enzymatic cues provided by TBK1 to locate and silence the IFN-I locus.

### UHRF2 hijacks nuclear translocated TBK1 for targeting type I interferon gene loci

**Figure 3:**
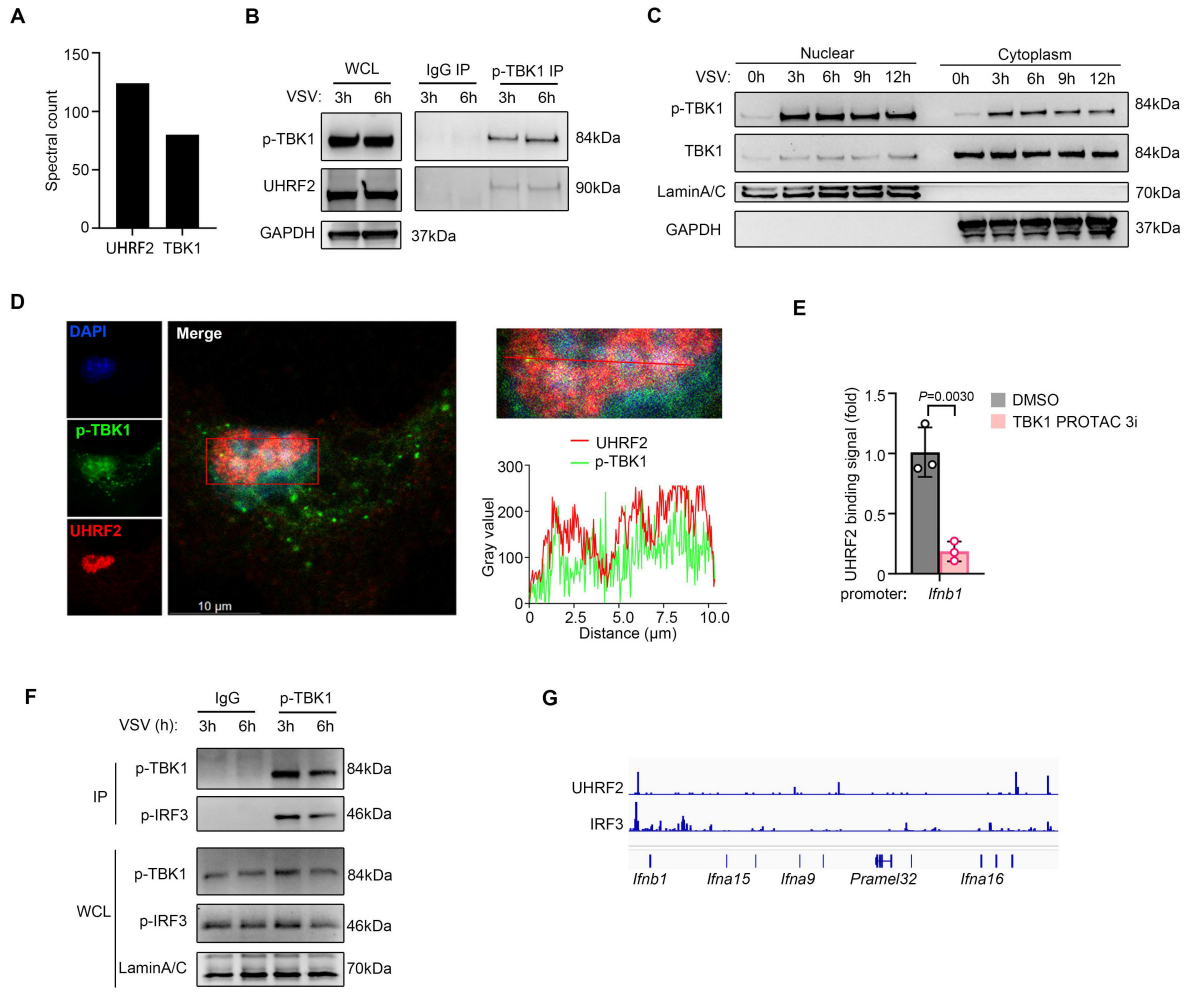
** UHRF2 interacts with nuclear TBK1 to specifically repress IFN-I expression.** (**A**) Co-immunoprecipitation (co-IP) of UHRF2 in total cell lysates of mouse macrophage for LC-MS/MS analysis. MS spectral count of indicated proteins which were not detected in IgG control group were listed. (**B, F**) Immunoblot analysis of endogenous indicated proteins in macrophages infected with VSV for 3 or 6 h, assessed before (whole-cell lysates (WCL); left) or after (IP; right) immunoprecipitation with IgG or antibody to phosphorylated TBK1 (p-TBK1). (**C**) Immunoblot analysis of phosphorylated (p-) or total TBK1 in nuclear or cytoplasm fractions of macrophages infected with VSV for indicated times. (**D**) Confocal microscopy of the co-localization of UHRF2 and p-TBK1 in macrophages infected with VSV for 3 h, and the intensity curve was plotted on the bottom. Scale bar, 10 μm. (**E**) ChIP-qPCR analysis of UHRF2 at the *Ifnb1* promoter in macrophages pretreated with TBK1 degrader (TBK1 PROTAC 3i) for 6 h and then infected with VSV for 12 h. (**G**) Normalized binding signals of indicated proteins in CUT&Tag assay using macrophages infected with VSV for 3 h. Data were presented as mean+s.d (**E**) or images (**B, C, D, F**) from one representative of three independent experiments with biological replicates.

To explore how a traditionally cytosolic kinase like TBK1 could facilitate nuclear chromatin targeting by UHRF2 for gene-specific regulation of IFN-I expression, we went further to investigate whether specific protein activated by anti-viral signaling was required for targeting of UHRF2 to IFN-I gene loci. We then performed co-immunoprecipitation (co-IP) assay coupled with liquid chromatography-mass spectrometry/mass spectrometry (LC-MS/MS) to map the physical interactome of UHRF2 in macrophages under infectious conditions. Interestingly, the interactome analysis yielded an unexpected and highly significant hit: TBK1 (**Fig. 3A**).

Virus sensor signaling activates TBK1 characterized with Ser172 phosphorylation in TBK1 (p-TBK1). We found that UHRF2 preferentially complexed with the p-TBK1 following viral infection (**Fig. 3B**). While TBK1 is canonical to the cytosol, where it interacts with adaptors like MAVS and STING. The nuclear localization of UHRF2 implied that anti-viral signaling might induce nuclear translocation of activated TBK1. We then isolated nuclear proteins of macrophages and indeed detected the nuclear translocation of both total TBK1 and p-TBK1 after VSV infection (**Fig. 3C**). High-resolution confocal assay confirmed these biochemical findings, demonstrating spatial colocalization of UHRF2 and p-TBK1 within the nucleus of infected macrophages (**Fig. 3D and Fig. S2A**). We then employed a highly specific proteolysis targeting chimera (TBK1 PROTAC 3i) to trigger the rapid, chemically induced degradation of TBK1 30. In macrophages pretreated with the TBK1 PROTAC, the virus-induced recruitment of UHRF2 to the *Ifnb1* promoter was significantly reduced (**Fig. 3E** and **Fig. S2B**). According to UniProt database, TBK1 lacks a DNA binding domain. TBK1 targeting IFN-I loci may depend on its binding partner like IRF3. We indeed detected the interaction between p-TBK1 and Ser396 phosphorylated IRF3 (p-IRF3) in the nuclear fraction of VSV-infected macrophages (**Fig. 3F**), also implying an involvement of the activated IRF3 in the nuclear translocation of TBK1. We further performed cleavage under targets and tagmentation (CUT&Tag) sequencing assay, and found colocalization of UHRF2 and IRF3 in IFN-I loci in VSV-infected macrophages (**Fig. 3G**). These data indicate that nuclear p-TBK1 acts as a scaffolding protein to facilitate IRF3-dependent recruitment of UHRF2 to IFN-I loci. Thus, UHRF2 completely compensates for its lack of sequence specificity by physically hijacking nuclear translocated, activated TBK1, using the kinase as a molecular bridge to specifically access the IFN-I gene loci and mediate gene-specific transcription repression.

### UHRF2 represses the expression of IFN-I through interacting with HDAC1

**Figure 4:**
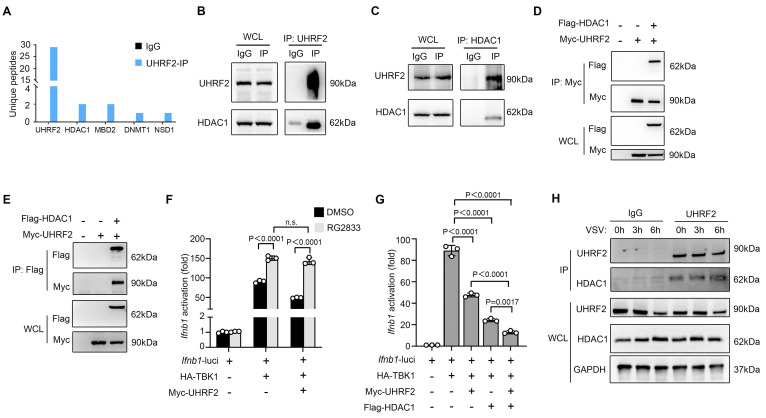
** UHRF2 represses the expression of IFN-I through interacting with HDAC1.** (**A**) Co-IP of UHRF2 in total cell lysates of BMDM for LC-MS/MS analysis. Unique peptides of indicated genes were listed. (**B, C**) Co-IP of UHRF2 and HDAC1 in total cell lysates of macrophages and immunoblot with indicated antibodies. Whole cell lysis (WCL) was used as input. (**D, E**) HEK293T cells were transfected with Myc-tagged UHRF2 and Flag-tagged HDAC1 for 48 h. WCL were IPed with anti-Myc antibody (**D**) or anti-Flag antibody (**E**), the interactions were examined by immunoblot with indicated antibodies. WCL were used to examine the input of overexpressed proteins. (**F, G**) Luciferase activity of the *Ifnb1* promoter reporter in HEK293T cells transiently transfected with indicated plasmids with or without RG2833 (50 nM) treatment and assessed by dual-luciferase assay. (**H**) Immunoblot analysis of endogenous UHRF2 or HDAC1 in macrophages infected with VSV for 0, 3, 6 h (above lanes), assessed before (WCL; lower) or after (IP; upper) co-IP with antibody to UHRF2. Data were presented as mean+s.d (**F, G**) or images (**B-E, H**) from one representative of three independent experiments with biological replicates. n.s., not significant.

On the basis of the above discovery that UHRF2 targets specifically to the IFN-I gene locus, we then investigated the epigenetic machinery it deploys to silence transcription of IFN-I. UHRF2 has been characterized to be a reader of 5-hydroxymethylcytosine (5hmC), a mark catalyzed by Ten-eleven translocation (TET) to promote DNA demethylation 21, 22. However, the predominant TET proteins in macrophages, TET2 and TET3, have been shown to regulate innate immunity through mechanisms entirely independent of their catalytic 5-hmC activity at IFN-I promoters, often functioning through recruitment of other repressors rather than direct mediating demethylation there 31-33. Thus, we hypothesized that UHRF2 relies on a 5-hmC-independent mechanism to execute gene repression. Returning to our LC-MS/MS interactome data, we identified the presence of several repressive chromatin modifiers bound to UHRF2, most notably histone deacetylase 1 (HDAC1) (**Fig. 4A**). HDAC1 is a well-established corepressor deeply involved in inhibiting inflammatory gene transcription by altering chromatin accessibility 10. We then validated the robust, direct interaction between UHRF2 and HDAC1 in primary macrophages in co-IP assay, validating the mass spectrometry data (**Fig. 4B, C**). To further confirm this, we then overexpressed Myc-tagged UHRF2 with Flag-tagged HDAC1 in HEK293T cells; reciprocal co-IPs demonstrated strong complex formation between the two proteins (**Fig. 4D, E**).

The functional relevance of this interaction in the context of interferon signaling was demonstrated using the specific HDAC1/3 pharmacological inhibitor RG2833. In luciferase reporter assays, in the presence of RG2833, overexpressed UHRF2 could not significantly repress TBK1-activated *Ifnb1* transcription compared with the control group, proving that HDAC1 enzymatic activity is strictly required for UHRF2-mediated silencing (**Fig. 4F**). Moreover, the overexpression of UHRF2 could further inhibit TBK1-activited promoter activity of *Ifnb1* when HDAC1 was overexpressed in HEK293T cells, suggesting a synergistic repressive relationship (**Fig. 4G**). Temporally, the interaction between endogenous UHRF2 and HDAC1 was significantly enhanced during the early hours of VSV infection, precisely coinciding with the assembly of the repressive complex at the chromatin (**Fig. 4H**). The redundancy among class I HDACs for epigenetic regulation is generally accepted. In reporter assays, we found that HDAC2 or HDAC3 could not act concurrently with UHRF2 to inhibit the promoter activity of *Ifnb1* although either HDAC2 or HDAC3 could inhibit TBK1-increased promoter activity of *Ifnb1* alone (**Figure S3**). These results indicate that UHRF2 selectively acts with HDAC1 to epigenetically repress the expression of IFN-I.

### UHRF2 stabilizes HDAC1 via K29-linked polyubiquitination to enforce repression

**Figure 5:**
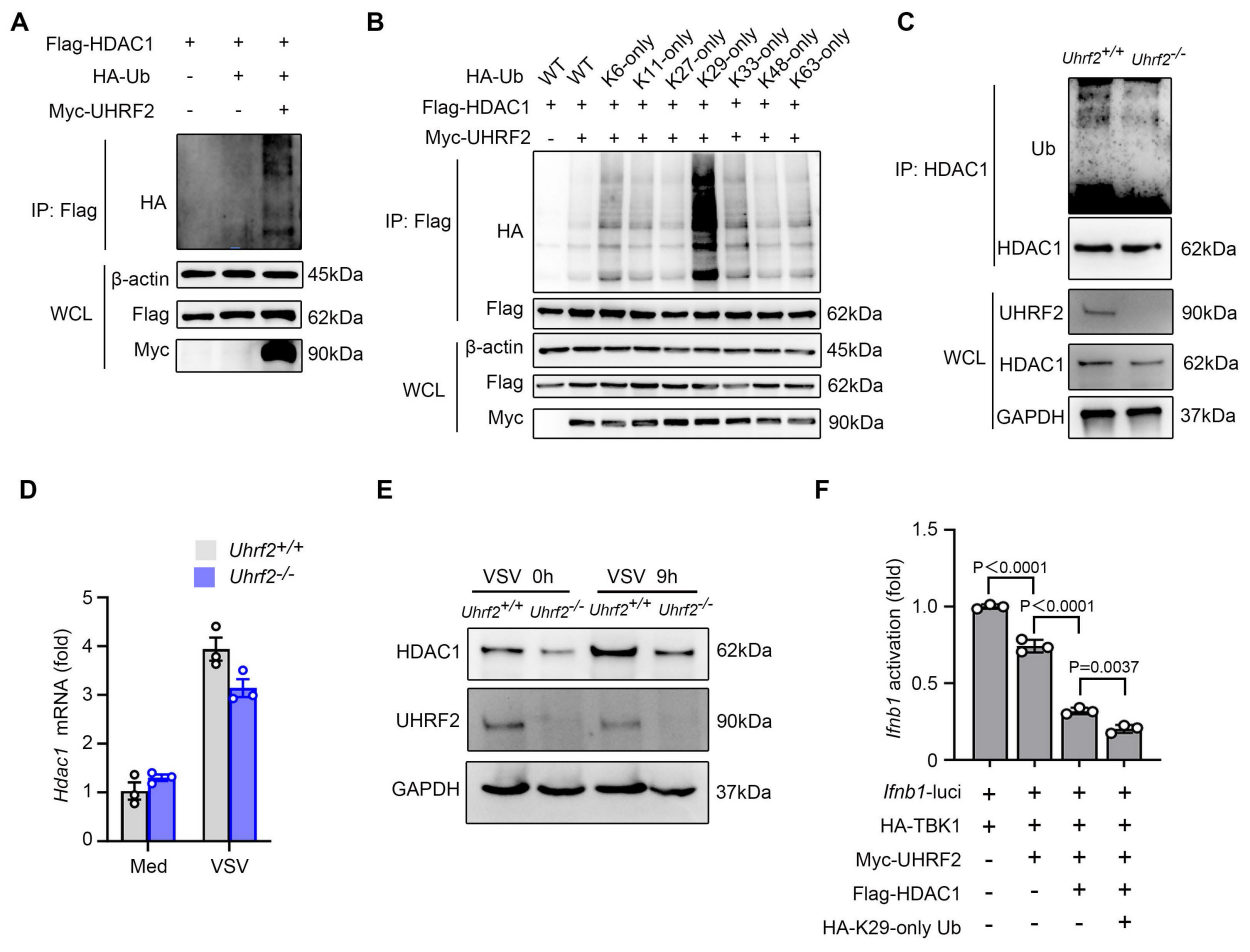
** UHRF2 stabilizes HDAC1 through mediating K29-linked polyubiquitination.** (**A**) HEK293T cells were transiently transfected with vectors encoding Flag-tagged HDAC1, Myc-tagged UHRF2 and HA-tagged wild Ub for 48 h. Then, polyubiquitination of immunoprecipitated Flag-HDAC1 was evaluated by immunoblot. (**B**) HEK293T cells were transiently transfected with Flag-tagged HDAC1, Myc-tagged UHRF2 and indicated HA-tagged Ub mutant vectors as indicated for 48h. Site specific polyubiquitination of immunoprecipitated Flag-HDAC1 was evaluated by immunoblot. (**C**) Immunoblot analysis of polyubiquitination of endogenous immunoprecipitated HDAC1 in *Uhrf2^+/+^* or *Uhrf2^-/-^* mouse macrophages infected with VSV for 12 h. (**D, E**) RT-qPCR analysis of *Hdac1* mRNA levels (**D**) and immunoblot analysis of HDAC1 protein levels (**E**) in *Uhrf2^+/+^* or *Uhrf2^-/-^* macrophages during VSV infection. (**F**) Luciferase activity of the *Ifnb1* promoter reporter in HEK293T cells transiently transfected with indicated plasmids, assessed by dual-luciferase assay. When performing polyubiquitination assay, MG132 was added 8 h before cell lysis. Data were presented as mean+s.d (**D, F**) or images (**A, B, C, E**) from one representative of three independent experiments with biological replicates.

UHRF2 possesses a highly conserved C-terminal RING finger domain, endowing it with intrinsic E3 ubiquitin ligase activity 19. We then investigated whether UHRF2 covalently modifies HDAC1 for its polyubiquitination. In cellular assays co-expressing Flag-tagged HDAC1 with Myc-tagged UHRF2 and HA-tagged ubiquitin, we observed that UHRF2 overexpression significantly increased the polyubiquitination of overexpressed HDAC1 (**Fig. 5A**). To determine the specific architecture of the ubiquitin chains, which dictates the functional outcome of the modification, we utilized a panel of ubiquitin mutants in which all lysines except one were mutated to arginine (e.g., K29-only, K48-only, K63-only). UHRF2 overexpression most significantly increased the assembly of K29-linked polyubiquitin chains on HDAC1 (**Fig. 5B**). Interestingly, the result showed less increased signal in WT ubiquitin group than that in K29-only ubiquitin group, probably due to the inhibitory role of specific types of ubiquitination of the binding partners of HDAC1 in K29 polyubiquitination of HDAC1. Moreover, we also observed significant decreases in the polyubiquitination of endogenous HDAC1 in *Uhrf2*-KO macrophages infected with VSV (**Fig. 5C**).

As reported, K29-linked ubiquitination maintains protein homeostasis 34. We then investigated that UHRF2 stabilized or destabilized HDAC1. We noticed that although MG132 was used in ubiquitination assays, overexpression of UHRF2 weakly increased and Uhrf2-KO decreased the protein levels of HDAC1 in the whole cell lysates controls (**Fig. 5A-C**). UHRF2 loss decreased protein but not mRNA levels of endogenous HDAC1 in macrophages especially after VSV infection (**Fig. 5D, E**). These results demonstrate that UHRF2 stabilizes HDAC1 through mediating K29-linked polyubiquitination of HDAC1. This absolute dissociation between mRNA and protein levels confirms that UHRF2 regulates HDAC1 purely at the post-translational level, preventing its turnover. We then investigated this regulatory mechanism in repressing the expression of IFN-I. Overexpressed Ub mutant (K29 only) could further promote the repressive effect of overexpression both UHRF2 and HDAC1 on TBK1-activated promoter activity of *Ifnb1* (**Fig. 5F**). Taken together, these data demonstrate a highly non-canonical mechanism in which the E3 ligase UHRF2 deploys atypical K29-linked polyubiquitination not as a degradation signal, but as a stabilizing force that extends the half-life of HDAC1 specifically at the target chromatin locus, ensuring sustained epigenetic repression.

### The stabilized UHRF2-HDAC1 axis terminates IFN-I transcription by erasing H4K12la

**Figure 6:**
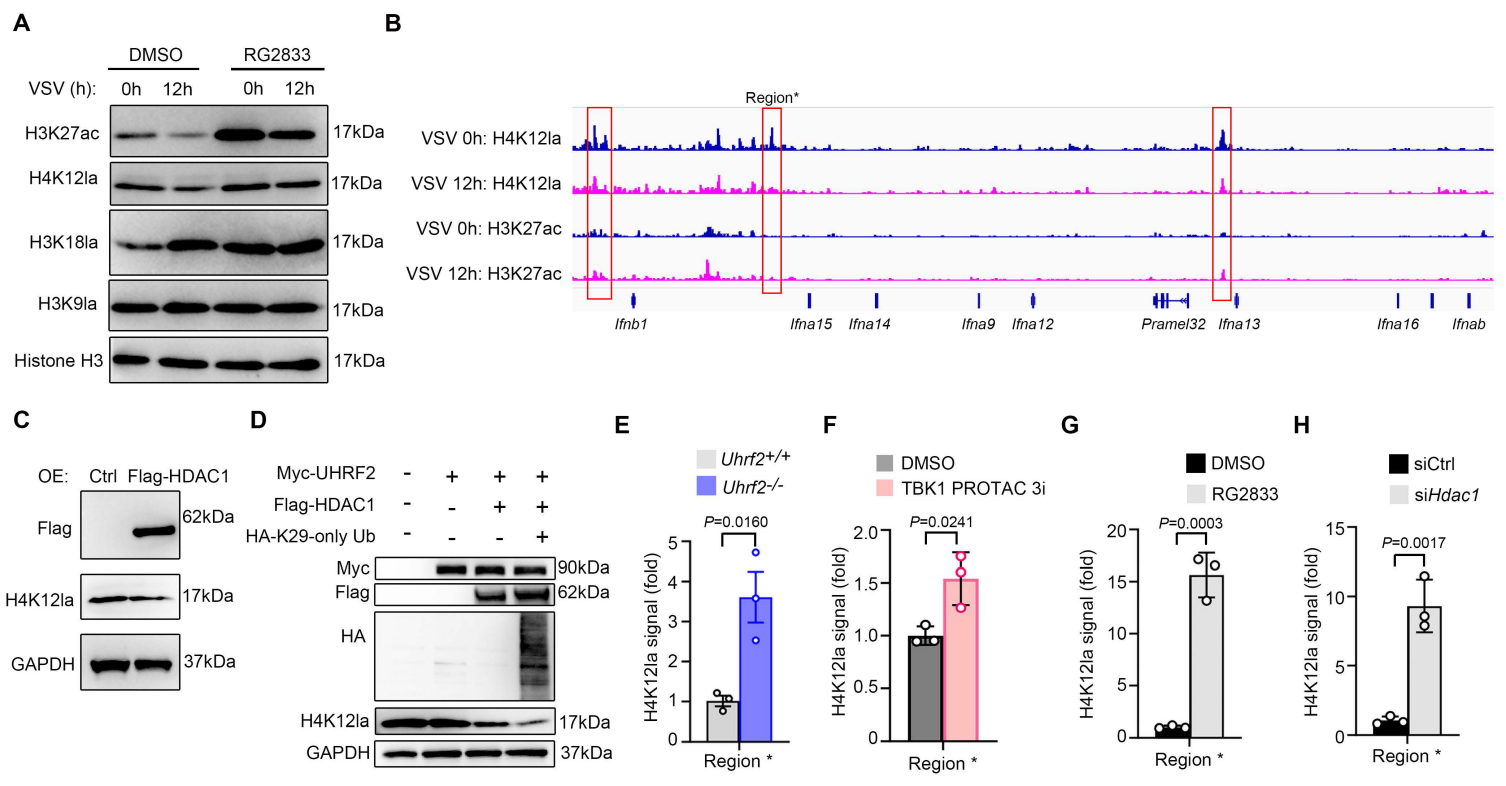
** UHRF2 represses IFN-I expression through HDAC1-mediated erasing H4K12la.** (**A**) Immunoblot analysis of indicated histone modifications in mouse macrophages infected with or without VSV, in the presence or absence of RG2833. (**B**) Signals of H4K12la and H3K27ac in IFN-I gene loci in macrophages infected with VSV for 12h in CUT&Tag analysis. Region* was used for ChIP-qPCR analysis in E-H. (**C**, **D**) Immunoblot analysis of H4K12la in the HEK293T cells overexpressed with indicated proteins. (**E**-**H**) ChIP-qPCR analysis of H4K12la enrichment in region* in either *Uhrf2^+/+^* or *Uhrf2^-/-^* macrophages (**E**), wildtype macrophages pretreated with TBK1 degrader for 6 h (**F**), wildtype macrophages treated with RG2833 (50 nM) (**G**) or wildtype macrophages silenced with* Hdac1* (**H**), and infected with VSV for 12 h. Data were presented as mean+s.d (**E-H**) or images (**A**, **C, D**) from one representative of three independent experiments with biological replicates.

Since HDACs can regulate histone acetylation and lactylation, we then investigated whether histone acetylation and lactylation were involved in UHRF2-HDAC1 axis-mediated repression of IFN-I transcription. Global immunoblotting of infected macrophages revealed that VSV infection selectively induced decreases in specific activating marks H3K27ac and H4K12la, and an increase in H3K18la. HDAC1/3 inhibitor RG2833 almost completely impaired VSV infection-induced decrease of the level of H4K12la but not H3K27ac (**Fig. 6A**). We further performed CUT&Tag sequencing analysis to investigate these two types of histone acylation, and found that VSV infection promoted H3K27ac level but inhibited H4K12la level in IFN-I gene loci (**Fig. 6B**), implying a repression role of erasing H4K12la in IFN-I gene transcription.

We wondered whether UHRF2-HDAC1 was involved in this repression mechanism. Overexpression of HDAC1 decreased total H4K12la in HEK293T cells (**Fig. 6C**). Furthermore, overexpression of HDAC1 could decrease H4K12la level in HEK293T cells overexpressed with UHRF2, especially with Ub mutant (K29 only) (**Fig. 6D**). Moreover, ChIP-qPCR analysis showed that *Uhrf2*-KO, TBK1 degrader, RG2833 as well as silencing *Hdac1* all increased H4K12la in IFN-I gene loci after VSV infection (**Fig. 6E-H and Fig. S4**). These results indicate that UHRF2 hijacks TBK1 to specifically repress the expression of IFN-I through promoting HDAC1-mediated erasing of H4K12la in IFN-I gene loci.

Since UHRF2 selectively inhibits IFN-I expression, UHRF2 may be also involved in type I interferonopathies, such as SLE. We reanalyzed the published scRNA-seq data of skin biopsies of SLE patients and healthy donors (HC) (GSE179633). Through clustering of cell subsets, we found that *UHRF2* had the highest expression in cluster 13 with CD4^+^ T cell-specific markers (**Fig. S5A-C**). Interestingly, although IFN-α are critical for development of SLE, we found *IFNB1* had the higher expression level than analyzed IFN-α among clusters in skin samples, which had highest expression in cluster 12 with myeloid dendritic cell (DC)-specific marker (**Fig. S5D**). We found that UHRF2 was expressed higher in the most of the SLE samples, compared with HC samples, in cluster 12 and cluster 13. The data indicated feedback upregulation of UHRF2 in immune cells of SLE for inhibiting type I interferon expression under the pathological condition type I interferonopathies, which was further evidenced by IFNB1 expression barely detected in the sample with the highest expression of UHRF2, and vice versa, in SLE group (**Fig. S5E, F**). These data suggest that expression variation of UHRF2 contributes to the abnormal expression of IFN-I in SLE.

## Discussion

The robust and rapid induction of type I interferons is a prerequisite for surviving acute viral challenge; yet, the failure to properly resolve this response will lead to tissue damage and autoimmune pathologies. IFN-I induction and expression are tightly regulated at multiple levels. There are both positive and negative regulators like Trim25, SOCSs and A20 which regulate anti-viral innate signaling to affect expression of IFN-I 35, 36. IFN-I expression is also regulated at transcription and post-transcription levels, e.g., Sirtuin 1 and RAD18 targeting IRF3/7 and SP140 destabilizing *Ifnb1* mRNA 37-39. In this study, we elucidate a multi-tiered regulatory network that coordinates the precise temporal and spatial resolution of the IFN-I response. We reveal that the E3 ligase UHRF2 functions as a highly specialized, gene-specific epigenetic repressor that directly links innate immune signal transduction to the epigenetic landscape of the host cell.

A central conceptual advancement of this work is the elucidation of an activation-induced repression model that relies on the repurposing of a canonical signaling kinase. Traditionally, the TBK1-IRF3 axis is viewed strictly as a cytosolic engine for signal amplification. Our identification of TBK1 within the nuclear compartment, physically tethered to UHRF2, redefines TBK1 as a bimodal protein. In the cytosol, TBK1 activates the transcription factors required to initiate immunity; however, upon translocating to the nucleus, activated TBK1 serves as a highly specific scaffold, guiding the repressive epigenetic machinery directly to the chromatin loci it just helped activate. Because UHRF2 lacks an intrinsic sequence-specific DNA-binding domain capable of DNA element-specific targeting, it is entirely reliant on the spatial cues provided by nuclear TBK1. This mechanism elegantly explains how broad epigenetic modifiers can achieve gene-specific targeting without the need for bespoke DNA-binding domains. Whether TBK1 translocates into the nucleus autonomously, piggybacks on the nuclear import machinery of IRFs, or relies on unique adaptor proteins remains an exciting avenue for future investigation.

The temporal dynamics of this system highlight a sophisticated evolutionary strategy for maximizing host survival. Based on our transcriptomic and genetic data, we propose a positive feedback loop governed by STAT1-mediated transcriptional repression. During the nascent stages of viral infection, the initial wave of IFN-I signaling actively represses the transcription of the UHRF2 gene through STAT1. By transiently removing the UHRF2-dependent epigenetic brake, the cell licenses a period of unhindered, massive interferon amplification. This guarantees that the host can quickly establish a systemic antiviral state ensuring the elimination of the invading viruses. However, along with the infection period extended to the late stage, the newly synthesized UHRF2 proteins hijack the pool of nuclear TBK1, rapidly assembling at the hyperactive IFN-I promoters to enforce a hard epigenetic shutdown, thereby timely and preciously controlling IFN-I levels to prevent inflammatory damage and autoimmune pathology.

Although UHRF2 acts as a 5-hmC reader to promote DNA demethylation, it was recently reported to be required for resistance to DNA methylation reprogramming in primordial germ cells 40, implying epigenetic regulatory role of UHRF2 beyond DNA demethylation. Our data showed that UHRF2 mediates K29-linked polyubiquitination of HDAC1 to stabilize it in cells, linking UHRF2 to histone deacylation. In our model, we identified a role of UHRF2 in promoting HDAC1 erasing H4K12la in IFN-I loci in a TBK1-IRF3 dependent manner. Moreover, UHRF2 may interact with other transcription factors and read chromatin modifications like 5-hmC, thus, UHRF2 may specifically target other gene loci for transcription regulation in addition to IFN-I, or be involved in regulating chromatin status beyond histone delactylation, which deserves further investigation.

Although class I HDACs (HDAC1-3) was reported to either erase or catalyze lysine lactylation 16, 41, assay of the HDAC inhibitor only showed increased levels of specific types of histone acetylation and lactylation in our model. Whether H4K12la is an inherent specific substrate of HDAC1 needs further investigation. Moreover, loss of H4K12la after VSV infection further indicates that erasing H4K12la plays an important role in activation-induced repression at epigenetic level during pathogen infection. At initial phase during retinoic-acid inducible gene I (RIG-I)-like receptor (RLR)-induction of IFN-I production, the level of lactate which inhibits MAVS aggregation is downregulated for boosting IFN-I induction 42. This may act as a feedback signal for promoting erasing H4K12la for supplementing lactate, which can in turn terminate RLR signaling. Thus, erasing H4K12la and the lactate derived from this process can coordinately terminate IFN-I expression at late phase of VSV infection.

At the biochemical level, our data uncover a post-translational dynamic: the utilization of K29-linked polyubiquitination to stabilize a nuclear corepressor. Although K29/K48-branched ubiquitin chains were reported to induce protein degradation 43, previous studies reveal K29-linked ubiquitination in maintaining protein homeostasis through a dual mechanism involving either protein degradation or stabilization 34. Our data further showed K29-linked polyubiquitination in stabilizing HDAC1 in nucleus. Since the regulation mechanism underlying maintaining homeostasis of nuclear proteins may be different from cytosolic proteins 44, the K29-linked ubiquitination-dependent mechanism for stabilizing nuclear proteins need further investigation.

In summary, we have defined a comprehensive molecular axis, spanning cytosolic kinase activation, nuclear translocation, atypical ubiquitination, and epigenetic remodeling, that strictly restrains type I interferon production. Targeting the UHRF2-HDAC1-H4K12la axis presents a highly precise therapeutic vulnerability. Inhibiting UHRF2 could artificially extend the early interferon amplification phase to combat refractory viruses, whereas enhancing its activity or stability may offer a novel strategy to epigenetically silence the pathological interferon signatures driving pathogenesis of autoimmune diseases.

## Materials and Methods

### Mice and *in vivo* viral infection models

C57BL/6 mice were obtained from Shanghai Laboratory Animal Center and used at the age of about 6-8 weeks. *UHRF2* knockout mice on a C57BL/6JGpt background and *Ifngr1/Ifnar1* double knockout mice were obtained from GemPhamatech Co., Ltd. All mice were maintained under specific pathogen-free conditions. All animal experiments were performed according to National Institute of Health Guide for the Care and Use of Laboratory Animals, with the approval of the Scientific Investigation Board of Naval Military Medical University, Shanghai. The *in vivo* VSV infection and survival assays were performed as previously described 13. Briefly, age- and sex-matched wild-type and *Uhrf2-KO* littermates were subjected to intraperitoneal injection with VSV (5 × 10^7^ PFU/g). Post-infection, tissues (liver, lung, spleen) were harvested at 24 hours. Viral loads in these tissues were quantified by RT-qPCR, and pathological changes were assessed by H&E staining.

### Reagents and antibodies

ChIP Grade Protein G Magnetic Beads (#9006) and Cell Lysis Buffer (#9803) were from Cell Signaling Technology. Primary antibodies were as follows: anti-GAPDH (1:2000; Cell Signaling Technology, 2118), anti-UHRF2 (1:200; Santa Cruz, sc-398953), anti-TBK1 (1:1000; Cell Signaling Technology, 3504), anti-p-TBK1 (1:1000; Cell Signaling Technology, 5483), anti-IRF3 (1:1000; Cell Signaling Technology, 4302), antip-IRF3 (1:1000; Cell Signaling Technology, 4947), anti-UHRF2 (1:200; Santa Cruz, sc-81598), anti-HDAC1 (1:200, Santa Cruz, sc-81598), anti-LaminA/C (1:2000; Cell Signaling Technology, 2032), anti-β-Tubulin (1:2000; Cell Signaling Technology, 2128), anti-β-Actin (1:5000; Cell Signaling Technology, 4967), Anti-DYKDDDDK Tag (1:2000; Cell Signaling Technology, 14793), Anti-Myc Tag (1:2000; Cell Signaling Technology, 2278), Anti-HA Tag (1:2000; Cell Signaling Technology, 2367), Anti-L-Lactyl-Histone H3 (Lys9) (1:1000; PTMab, PTM-1419RM), Anti-L-Lactyl-Histone H3 (Lys18) (1:1000; PTMab, PTM-1427RM), Anti-L-Lactyl-Histone H4 (Lys12) (1:1000; PTMab, PTM-1411RM), Anti-H3K27ac (1:1000; Cell Signaling Technology, 8173), Anti- Histone H3 (1:1000; PTMab, PTM-1001RM). The secondary antibodies were antimouse IgG-HRP (1:2,000; Cell Signaling Technology, 7076), antirabbit IgGHRP (1:2,000; Cell Signaling Technology, 7074), Anti-rabbit IgG (Conformation Specific)-HRP (1:2,000; Cell Signaling Technology, 5127). RG2833(HY-16425), TBK1 degrader (HY-112557) and MG-132 (HY-13259) were purchased from MedChemExpress. Mouse IFNβ (12400) was purchased from PBL Assay Science.

### Isolation of peritoneal macrophages

Peritoneal macrophages were collected from mice 4 days post-injection (thioglycolate, BD). For lung macrophage isolation, mice were perfused with PBS and lung tissues were excised. Lung tissue digestion was performed with collagenase A (1 mg/ml) and Dnase I (1 unit/ml) for 45 min at 37 °C. Digested cell suspensions were filtered and lymphocyte-containing fractions were isolated using differential Percoll centrifugation.

### Cell culture and treatment

Peritoneal macrophages were cultured in RPMI1640 medium containing 10% FBS. HEK293T cells were cultured in DMEM medium supplemented with 10% FBS. All cells were cultured under 37 °C, 5% CO_2_ conditions. For infection assays, cells were exposed to VSV (1 M.O.I) for the indicated hours, and cytokine production was analyzed 12 hours post-infection.

### Reverse-transcription and quantitative PCR (RT-qPCR)

Total cellular RNA was extracted using TRIzol reagent (Invitrogen) and synthesized cDNA with the ReverTra Ace qPCR RT Kit (TOYOBO). Real-time PCR amplification was performed on a LightCycler System (Roche) using SYBR Green PCR Master Mix (Applied Biosystems) according to the manufacturer's instructions. Gene expression levels were normalized to *Gapdh* in each individual sample, and relative expression was calculated using the 2^-∆∆Ct^ method.

### Co-immunoprecipitation and immunoblot

Proteins interacted with UHRF2 were pull-down for LC-MS/MS or immunoblot analysis the way similarly as previously described 45. For Co-IP, antibody anti-Flag, anti-Myc, anti-UHRF2, anti-HDAC1, and anti-pTBK1 were used. Isolation of macrophage cytoplasm and nucleus fraction were performed using Nuclear and Cytoplasmic Protein Extraction Kit (Beyotime) in accordance with manufacture's instruction. The blot was developed using SuperSignal West Femto Maximum Sensitivity Substrate (Thermo Fisher).

### Protein polyubiquitination assay

Detecting the polyubiquitination of specific protein was performed as previously reported 46. The cells were lysed in the lysis buffer with 1% SDS. Lysates were denatured in 95 °C for 1 minute, followed by IP with specific antibody and protein G beads. The pellets were washed extensively four times with IP wash buffer. The final pellets were resolved in SDS sample buffer, and the polyubiquitination of specific protein was examined by immunoblot with specific antibodies.

### ChIP assay

The cells were treated with 1% (vol/vol) methanol-free formaldehyde for 10 min and processed as previously reported 31. Antibody-chromatin complexes were pulled-down using magnetic protein G beads (Cell Signaling Technology), following sequential washing steps, and subsequently eluted. After cross-link reversal and digestion with proteinase K, immunoprecipitated DNA was extracted via phenol-chloroform and ethanol precipitated. All data were normalized to the corresponding input DNA control.

### Immunofluorescent confocal microscopy

Immunofluorescence staining and confocal imaging were performed as described previously 47. For transfection experiments, HEK293T cells plated on glass coverslips were co-transfected with indicated plasmids, followed by immunolabelling with tag-specific antibodies. For endogenous protein detection, peritoneal macrophages plated on glass coverslips were stained with antibodies against UHRF2 or p-TBK1. All images were acquired using a Zeiss LSM910 confocal laser microscopy under a ×63 oil objective.

### Plasmid constructs and transfection

cDNA encoding mouse HDAC1, HDAC2, HDAC3, UHRF2 or TBK1 was amplified from peritoneal macrophages. They were cloned into pcDNA3.1-Flag, pcDNA3.1-Myc, or pcDNA3.1-HA eukaryotic expression vectors respectively. Each construct was confirmed by sequencing. Plasmids were transiently transfected into HEK293T cells with jetPEI reagents (Polyplus Transfection) according to the manufacturer's instructions.

### Luciferase reporter assay

HEK293T cells were co-transfected with *Ifnb1* luciferase reporter plasmid, pRL-TK-Renilla-luciferase plasmid, and other indicated vectors, with total DNA amounts equalized using empty control vector. After 24 hours, cells were lysed with Passive Lysis Buffer (Promega), and luciferase activities were measured using the Dual Luciferase Reporter Assay System (Promega) according to the manufacturer's protocol. Firefly luciferase activity was normalized to Renilla luciferase activity to correct for transfection efficiency.

### CUT&Tag sequencing

CUT&Tag experiment was performed using Hyperactive Universal CUT&Tag Assay Kit for Illumina (TD903, Vazyme, China), according to the standard protocol without modification. The libraries were sequenced using Illumina platform. 150bp pair-end reads with adaptors were trimmed and quality filtered by Trim_Galore, and aligned to mm10 reference using Bowtie2 48, 49. SAM file was filtered by Sambamba for removing duplicates based on uniquely mapped reads 50. A normalized bigwig file was generated using DeepTools for genome browser view 51.

### Statistical analysis

The data statistic was performed using Prism software (version 8.4.1, GraphPad). In experiments with only two groups, the unpaired Student's t test (unpaired and two tails) was used. One-way ANOVA with multiple comparisons was performed for experiments with more than two groups. Kaplan-Meier assay and Log-rank test was used for survival analysis.

## Supplementary Material

Supplementary figures.
