# Supplementary material for: E3 ligase UHRF2 hijacks nuclear TBK1 to epigenetically repress type I interferons expression
Source: Int J Biol Sci. 2026 May 18;22(10):5385–98. doi: 10.7150/ijbs.135125 (PMC13215362; doi:10.7150/ijbs.135125)
Supplement: Supplementary file 1 — Supplementary figures. [file ijbsv22p5385s1.pdf]

Supplementary Materials for

E3 ligase UHRF2 hijacks nuclear TBK1 to epigenetically repress type I interferons expression

Wenwen Huang *et al.*

Corresponding author email: qianzhang@immunol.org; caoxt@immunol.org

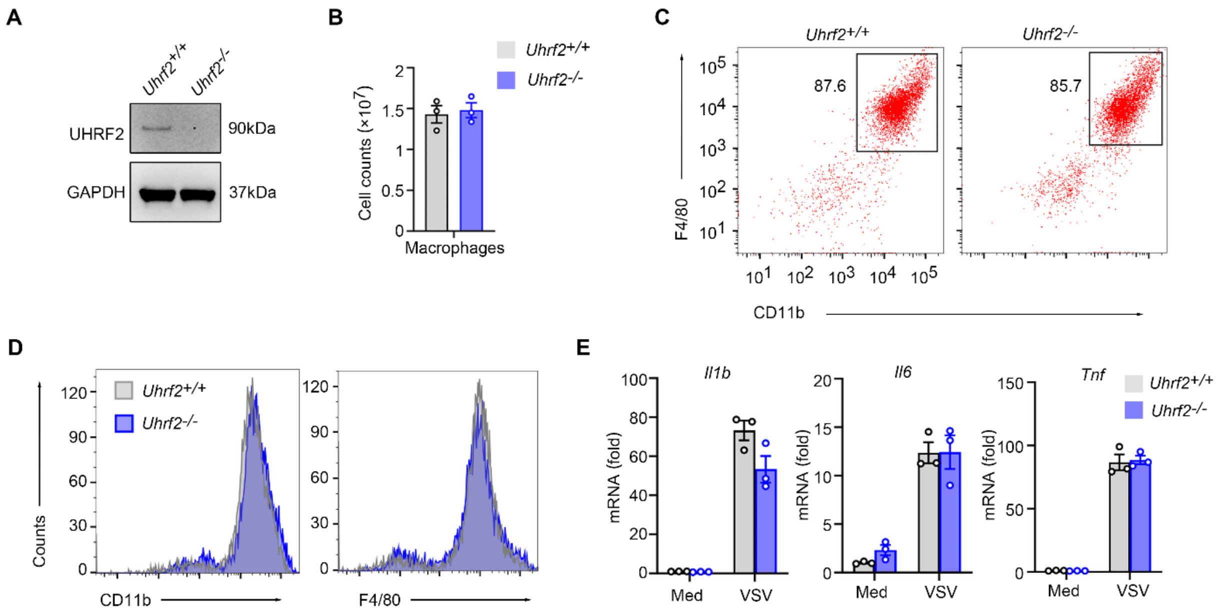

Fig. S1. *Uhrf2* knockout mice have no abnormal differentiation of macrophages. (**A**) Immunoblot analysis of UHRF2 expression in mouse peritoneal macrophages from *Uhrf2* knockout (*Uhrf2*$^{-/-}$) and control littermate (*Uhrf2*$^{+/+}$) mice. (**B**) Cell counts of peritoneal macrophages in *Uhrf2*$^{+/+}$ and *Uhrf2*$^{-/-}$ mice. (**C**, **D**) FACS analysis of CD11b, F4/80 expression in *Uhrf2*$^{+/+}$ and *Uhrf2*$^{-/-}$ peritoneal macrophages. (**E**) RT-qPCR analysis of mRNA levels of indicated genes in *Uhrf2*$^{+/+}$ or *Uhrf2*$^{-/-}$ macrophages infected with VSV for 12 h. Data were presented as mean+s.d (**B, E**) or images (**A, C, D**) from one representative of three independent experiments with biological replicates.

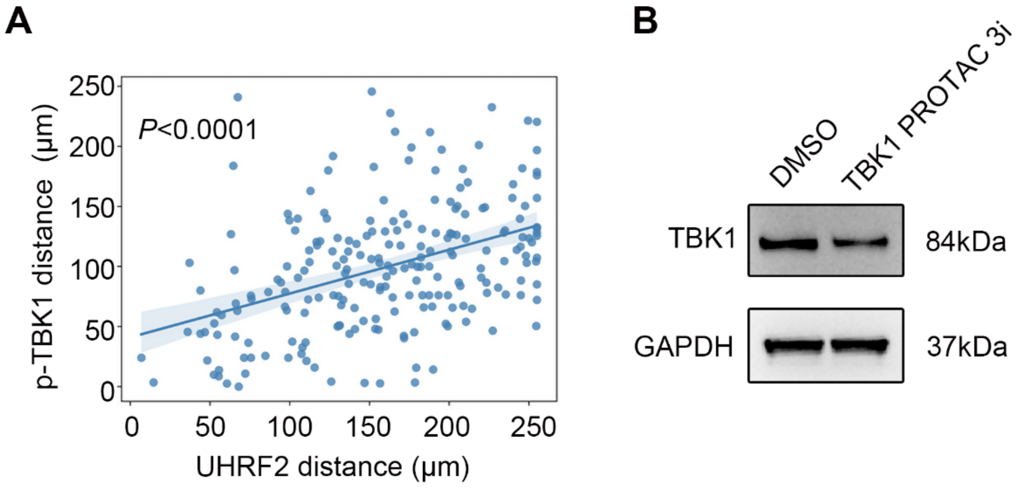

Fig. S2. Inducing degradation of TBK1 protein in macrophages. (**A**) Pearson correlation analysis of co-localization of UHRF2 and p-TBK1 (n=217). Statistical significance was determined using a two-tailed Pearson correlation test (Pearson r=0.4241, $P<0.0001$). (**B**) Immunoblot assays of total TBK1 protein in lysates of mouse macrophages pretreated with DMSO or TBK1 degrader (TBK1 PROTAC 3i) for 6 h and infected with VSV for 12 h. Data were presented as images (**B**) from one representative of three independent experiments with biological replicates.

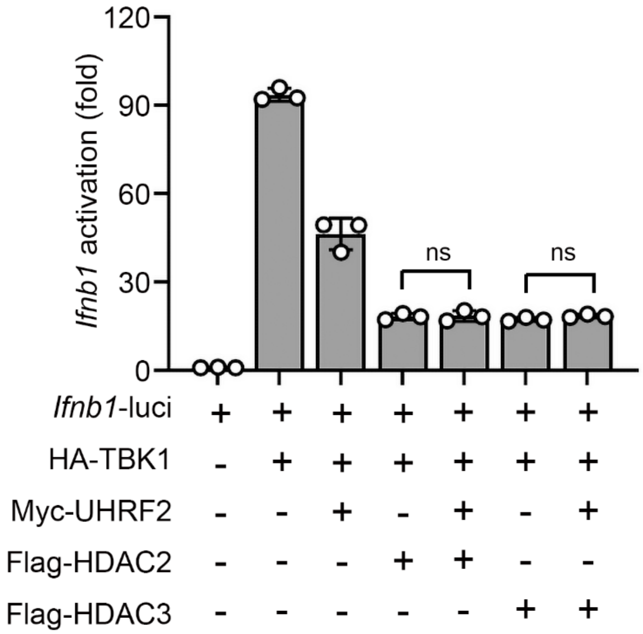

Fig. S3. HDAC2/3 independently inhibit TBK1-activated *Ifnb1* promoter activity. Luciferase activity of the *Ifnb1* promoter reporter in HEK293T cells transiently transfected with indicated plasmids and assessed by dual-luciferase assay. Data were presented as mean+s.d from one representative of three independent experiments with biological replicates.

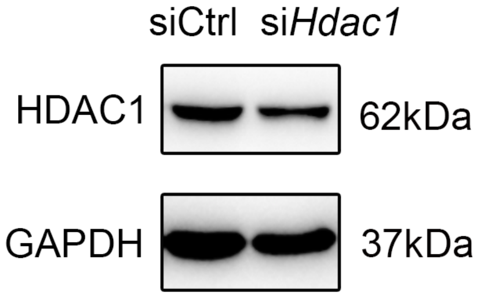

Fig. S4. Silencing *Hdac1* expression in macrophages. Immunoblot assays of HDAC1 in lysates of mouse macrophages transfected with control or *Hdac1*-specific siRNAs for 36 h and infected with VSV for 12 h. Data were presented as images from one representative of three independent experiments with biological replicates.

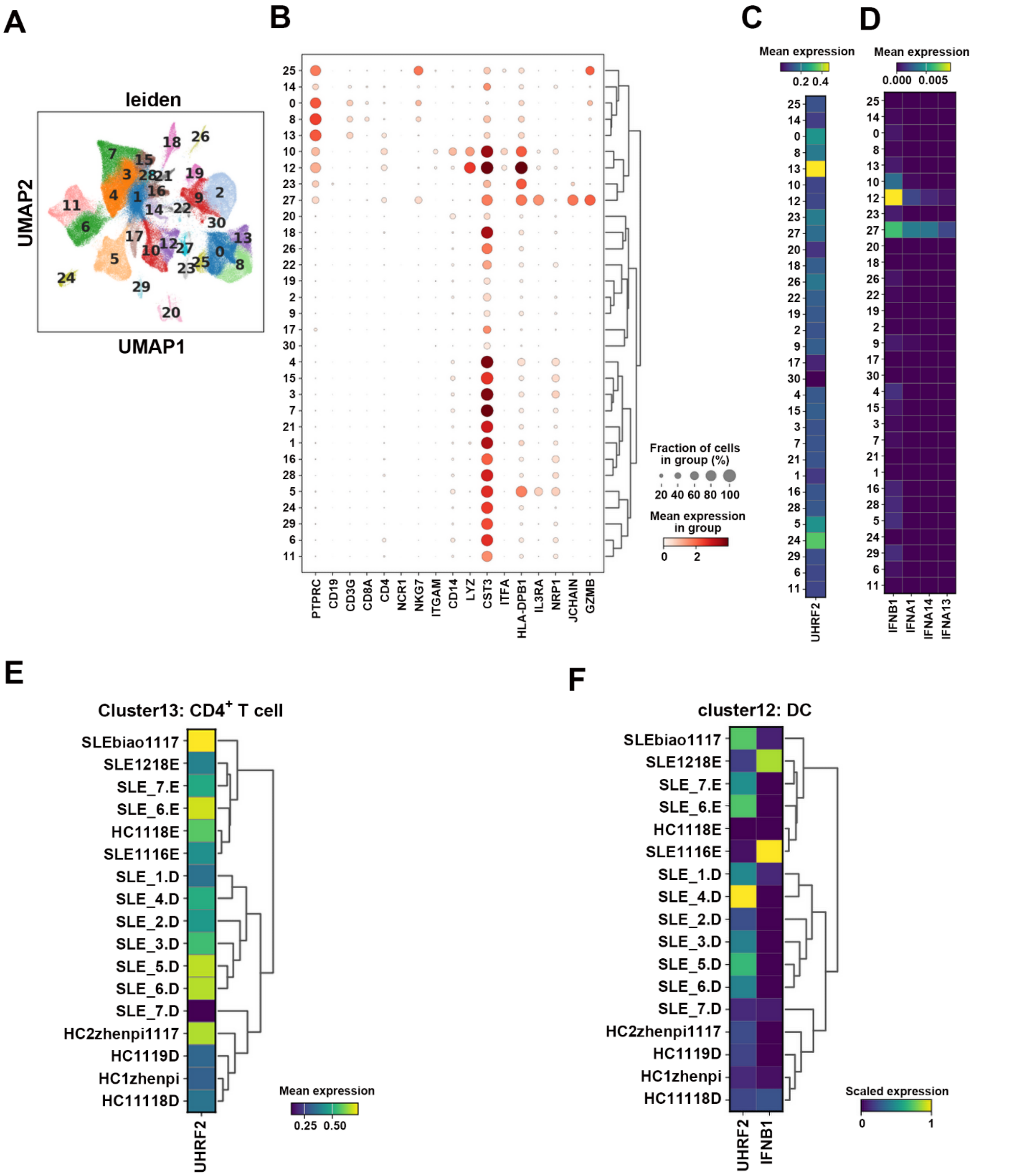

Fig. S5. Expression variations of UHRF2 in SLE samples. (**A**) UMAP of cell subsets in skin biopsies from SLE patients (SLE) and healthy donors (HC) in a published scRNA-seq dataset (GSE179633). (**B**) Dotplot showing mRNA levels of indicated genes in each of the cell clusters.

(**C-F**) Matrixplots showing mRNA levels of indicated genes in all clusters (**C, D**) or the indicated clusters in all the skin samples of the two groups (**E, F**).
